# Impact of the use of the internet on quality of life in older adults: review of literature

**DOI:** 10.1017/S1463423620000584

**Published:** 2020-12-02

**Authors:** Bhumika Aggarwal, Qian Xiong, Elisabeth Schroeder-Butterfill

**Affiliations:** 1 Msc Gerontology, University of Southampton, Southampton, UK; 2 Health Research, Lancaster University, Lancaster LA1 4YG, UK; 3 Centre for Research on Ageing Social Sciences University of Southampton, Southampton SO17 1BJ, UK

**Keywords:** elderly, information technology, internet, older adults, quality of life

## Abstract

**Aim:**

Given the paucity of data on the use of internet and quality of life (QoL), this literature review aimed to identify the motivations and barriers for internet use and the impact on QoL on older adults using the internet.

**Background:**

Even though older adults are increasingly using information technology, the numbers remain quite small globally. Currently published research primarily focuses on the various ways and methods of information technology use by older adults and the factors influencing use rather than on the impact of information technology on QoL of older adults.

**Methods:**

The studies included in this literature review were searched in three databases: WEB of Science, GoogleScholar and PubMed. English language articles were searched using the terms ‘older’, ‘elderly’, ‘senior’, ‘well-being’, ‘life satisfaction’, ‘quality of life’, ‘internet’ and “computer”.

**Findings:**

The review demonstrated the association of internet use on QoL in older adults. The majority of the studies substantiate the advantages of internet use by older adults including the ability to communicate with family and friends, maintain a wide social network, have access to information and participate in online leisure activities. There are some studies, though less in number, which did not find a relationship between well-being and use of internet by older adults. The policy implications of this review advocate a multidimensional strategy to support internet use by the older people incorporating internet training and education, financial issues, technical support and access needs to be developed.

## Introduction

As the population is ageing, there has been an increasing focus on a better quality of life (QoL), and the concept of active ageing has started to generate interest amongst researchers, academicians and policy-makers. The Madrid International Plan of Action on Ageing (2002) highlighted the role of technology use by older adults and indicated that having access to the internet and being able to use it could potentially help to decrease the feelings of loneliness, insignificance and the intergenerational disparity (MIPAA, United Nations, New York, 2002). The older adults globally have been resistant to adopt technology compared to the younger generations, but this trend is changing slowly. A growing number of older adults are overcoming the barriers to learn, use and keep updated with the information and communication technologies like internet, and this has only been facilitated with the availability of personal computers, cell phones, laptops, tablets and other devices. A growing number of older adults are overcoming the barriers to learn, use and keep updated with information and communication technologies like the internet, and this has been facilitated by the availability of personal computers, cell phones, laptops, tablets and other devices.

WHO defines QoL as ‘the “individuals” perception of their position in life in the context of the culture and value systems in which they live and in relation to their goals, expectations, standards and concerns’ (WHOQoL, 1998). The determinants of QoL for the older adults commonly include individual factors such as health, physical activity, socio-economic stability and personal control, and network factors such as social life, relationship with family, a care network and support system (Walker and Mollenkopf, 2007). The role of technologies including the internet in influencing QoL of the older adults has generally been a less explored theme globally and specifically in developing countries. With the increasing use of technology in everyday life, understanding the role of the internet on the QoL of the older population is important.

This literature review intends to explore the impact of the use of the internet on the quality of health of older adults and discusses the published evidence in relation to the impact of internet use on the older adults specifically in terms of QoL and tries to point out the gaps in current literature.

## Search methodology

**Figure 1.**
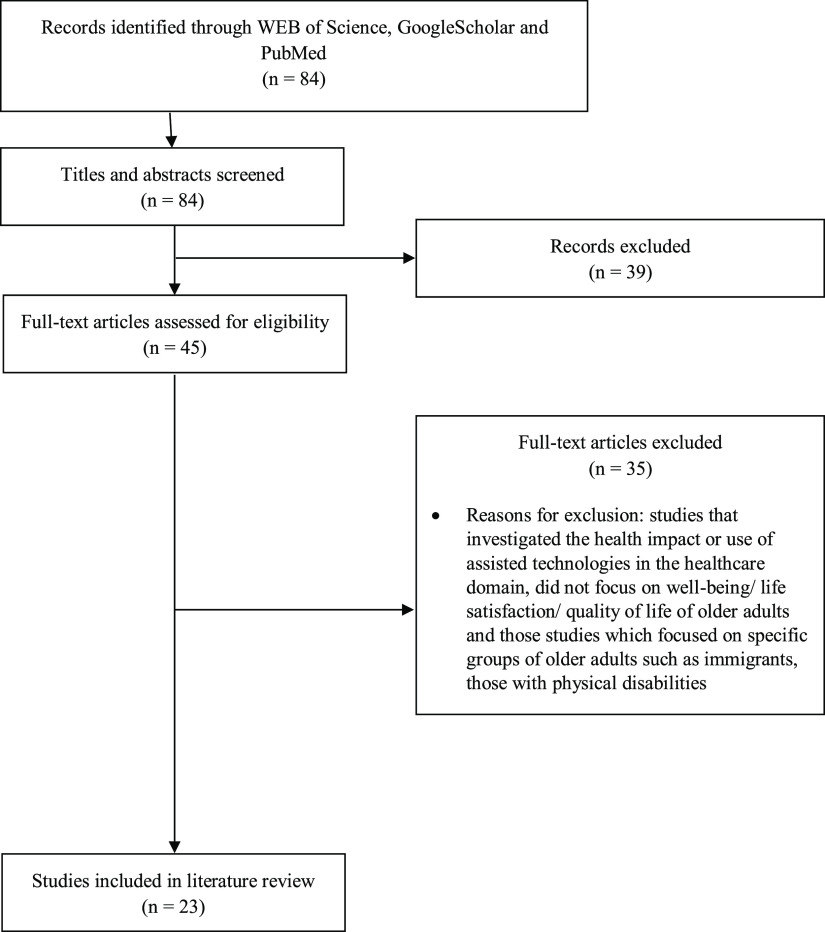
Flow diagram of the selection of studies

The currently published literature on the use of the internet by the older adults explores many broad areas including health, social, educational and psychological science. The studies included in this literature review were searched in three databases: WEB of Science, GoogleScholar and PubMed. English language articles were searched using the terms ‘older’, ‘elderly’, ‘senior’, ‘well-being’, ‘life satisfaction’, ‘quality of life’, ‘internet’ and ‘computer’ (Figure 1). Age ranges were not specified because of the wide variation of age ranges across studies. The date range was limited to a time period from 1 January 2000 to 30 December 2018. In total, 45 full-text articles were accessed, which examined the impact of internet use on the QoL of older adults. The studies that investigated the health impact or use of assisted technologies in the healthcare domain did not focus on well-being/life satisfaction/QoL of older adults, and those studies which focused on specific groups of older adults such as immigrants those with physical disabilities were excluded from the review considering the differences associated with using the internet compared with those without physical disabilities. Eventually, 38 articles were reviewed for relevance to this particular article and 23 articles were included for discussion in the literature review (Table 1).

**Table 1.** Summary of studies discussed in the literature review*

Reference	Country/age group	Methodology	Outcomes
Brown (2004)	Texas, USA/50 years of age and above	*n* = 60; experimental and control group, quantitative	Life satisfaction and self-esteem similar between the two groups
Chaumon *et al.* (2014)	France/mean age 87 years	*n* = 17, longitudinal study, effect of training and qualitative approach	Improved QoL; better social connectedness and communication
Chen and Persson (2002)	Ohio, USA/60 years and above	*n* = 218; comparison between older internet users and non-users and with younger adults; quantitative	Older internet users had better psychological well-being, specifically personal growth and sense of life purpose
Dorin (2007)	Kansas, USA/55 years of age and above	*n* = 93; comparison of internet users and non users; quantitative	Life satisfaction similar between the two groups
Elliot *et al.* (2013)	USA/65 years of age and above	*n* = 6443; cross-sectional survey; National Health and Ageing Trends study	No effect of use of information and communication technology on well-being or mental health
Erickson and Johnson (2011)	Canada/60 years of age and above	*n* = 122; cross-sectional survey; quantitative	Use of the internet correlated with wellness (loneliness and life satisfaction)
Firth and Mellor (2009)	Australia/65 years of age and above	*n* = 8, quantitative and qualitative	No difference in quantitative scores; qualitative interviews showed a sense of improved well-being
Hendrix (2000)	USA	Review	Improved self-esteem and social interaction
Hernandez-Encuentra *et al.* (2009)	Barcelona, Spain/65 years of age and above	*n* = 713; qualitative and quantitative	Information and communication technology linked to its usefulness, maintenance of independence.
Ihm and Hsieh (2015)	Chicago, USA/60 years of age and above	*n* = 1780; quantitative	Information and communication technology linked to well-being
Karavidas *et al.* (2005)	Florida, USA/53 years of age and above	*n* = 222; quantitative cross-sectional survey in older adults using computers	Increased self-efficacy and lower life satisfaction.
Koopman-Boyden and Reid (2009)	New Zealand/65 years of age and above	*n* = 1680, random sample, used WHOQoL and quantitative survey	Positive relationship between internet use and well-being, higher self-reported health status, and increased satisfaction with leisure and recreation activities.
Lelkes (2012)	Europe/65 years of age and above	*n* = 11,000; European Social Survey (ESS1); cross-sectional, quantitative	Better well-being, life satisfaction; less social isolation in older internet users
Mellor *et al.* (2008)	Australia/55 years of age and above	*n* = 20; 12-month study; quantitative and qualitative	Quantitative showed a negative impact, while qualitative results showed positive impact on well-being
Nimrod (2009)	USA	40 online communities, quantitative survey	Increased social support, self-preservation, leisure activity and social network
Osman *et al.* (2005)	UK/mean age 61 years	*n* = 50; Care OnLine project; qualitative interviews	Positive impact of internet training and use
Shapira *et al.* 2007)	Israel/70 years of age and above	*n* = 22; quantitative surveys	Older adults who began using the internet felt less depressed and more satisfied with life.
Slegers *et al.* (2008)	The Netherlands/65 years of age and above	*n* = 191; 12-month study; randomized control; SF-36 used; quantitative	Using computers and the internet did not influence daily functioning, well-being and social network of older adults.
Sum *et al.* (2008)	Australia/55 years of age and above	*n* = 222; online survey; quantitative	Lower levels of loneliness with use of the internet
Werner *et al.* (2011)	USA/60 years of age and above	*n* = 460; quantitative	Positive impact on well-being in older adults using the internet
White *et al.* (2002)	USA/60 years of age and above	*n* = 103; 5-month study; randomized control; quantitative and qualitative	Internet use associated with less loneliness and depression and more positive attitudes
Wright (2000)	USA/62 years of age and above	*n* = 136; online questionnaire; quantitative	Higher feeling of social support and satisfaction with internet use
Xie (2007)	China/60 years of age and above	*n* = 33; interview; grounded theory methodology; qualitative	Internet use made the post-retirement period more meaningful, improved self-evaluation and positively impacted with the well-being

* In the quantitative studies, non-validated measures were used such as researcher-selected questions or Likert scales.

## Review of literature

This review looks at published literature related to use of the internet by older people and the impact on their QoL. The articles of relevance are discussed after grouping them into subsections entitled: internet use: communication and social connectedness; internet use: information and services; and internet use: impact on well-being. There is a section on critical analysis and a table summarising the reviewed studies. Finally, the literature review concludes with a critical analysis and summary of the literature review and gaps in the currently available literature.

The studies on older adults and the internet can be broadly segmented into exploring the motivations and barriers of older adults’ use of computer technology, identifying differences in computer learning and usage across age groups, and the attitudes, benefits, and perceptions of using computers and the internet by older adults. There is no consensus in the currently published literature on the impact of use of information and communication technology on older adults. Many published studies highlight the positive effect of computer use on the psychological functioning and well-being of the older adults (Shapira *et al.* 2007; Erickson and Johnson 2011; Werner *et al.*, 2011). The use of the internet by older adults has also been associated with decreased loneliness and depression, better social connectedness, self-esteem and cognitive functioning (White *et al.*, 2002; Czaja and Lee, 2007), improved self-efficacy, self-control, self-determination, social interaction, education and skills development in older adults using the internet (Hendrix, 2000) in various studies. In contrast, there are other studies which did not find a correlation between use of the internet by the older adults and well-being or life satisfaction (Brown, 2004; Slegers *et al.*, 2008)

Per published literature, the primary activities for which the older adults use the internet include accessing e-mails (Koopman-Boyden and Reid, 2009), education, seeking information (Xie and Bugg, 2009) and shopping (Hernandez-Encuentra *et al.*, 2009). Kim (2008) in their review summarised the reasons for older adults to use the internet to be in contact with family and friends, to make new contacts and preserve social links, gather information related to current affairs, goods, services and healthcare, do online shopping, banking and leisure activities (Kim, 2008). The attitude and perception of older adults towards the use of the internet have been shown to be influenced by their education and socio-economic status (Koopman-Boyden and Reid, 2009). The benefits of using information technology by older adults include personal and social development and maintaining social relationships (Koopman-Boyden and Reid, 2009). This has given birth to the term ‘silver surfers’, defining the older adults using information and communication technology to narrow the intergenerational gap. Despite this, perceived barriers to the use of the information and communication technology amongst older adults exist. This could be due to various factors including the difficulties in learning new skills, high costs and lack of confidence in the use of technology (Goodall *et al.*, 2010).

### Internet use: communication and social connectedness

Two major themes emerged from the studies reviewed. The first was the impact of internet use by older adults is on communication with family and friends. The second was social connectedness including maintaining and building social support networks. Communication and social connectedness using the internet potentially impacts the QoL of older adults. White *et al.* (2002) looked at the psychological and social impact of the use of the internet on older adults (*n* = 100). Participants were randomised to two groups, the intervention group which received training on how to use the internet for 5 months and the control group. The intervention group had lower tendency of loneliness and depression, better communication and improved social interactions and a positive attitude towards the use of the internet in comparison to the group which did not receive training on using the internet. The findings were corroborated by the qualitative interview phase of the study (White *et al.*, 2002). The study focused on impact of internet training in older adults and not on the use of internet in older adults, and this is important when looking at the results of the study.

Increasingly today there is a growing population of younger generations moving away from home for work or education and the numbers of nuclear families is increasing. Being able to communicate with family and friends living away is one of the benefits of using the internet for older adults. In another study, it has been found that the use of the internet promotes the interactions of the older adults with family and friends and helps to expand their social network. Using the internet to communicate resulted in a positive impact on the loneliness and well-being of older adults. The authors found that in their study older adults using the internet had lower levels of loneliness, specifically social loneliness and better well-being. (Sum *et al.*, 2008). The authors found that in their study, older adults using the internet had lower levels of loneliness (particularly social loneliness) and better well well-being. (Sum *et al.*, 2008).

### Internet use: information and services

Using the internet empowers older adults to be able to access information related to news and current affairs, health-related updates, travel and leisure. Karavidas *et al.* (2005) found a positive impact on 222 older adults using the internet on life satisfaction due to various factors including feeling independent, being able to maintain their social networks and having access to information including health-related information. Using the internet for online shopping, banking, playing games and learning helped the older adults to manage even if they have impaired physical abilities (Shapira *et al.*, 2007). Participation in leisure activities and social networks using the internet can potentially increase the well-being of older adults (Nimrod, 2009). Similar findings were also seen in another study, in which the use of internet by older adults was a leisure activity possibly leading to a sense of well-being (Heo *et al.*, 2011).

### Internet use: impact on well-being

Well-being can be defined as the presence of positive emotions and moods including contentment and happiness, the absence of negative emotions (such as depression and anxiety), satisfaction with life, fulfilment and positive functioning (Ryff and Keyes, 1995). Wright (2000) found that in 136 older adults’ participation in online support groups had a positive impact on their psychological well-being, stronger social relations and better support networks in the older adults. It has been shown that use of technology is directly linked to a feeling of independence in older adults leading to a perception of better QoL (Mynatt and Rogers, 2002). Similar findings were seen in another study, where older adults using the internet were found to have higher psychological well-being levels than those not using the internet (Chen and Persson, 2002). The authors did note that the baseline scores in this study were high to begin with (Chen and Persson, 2002). Fifty older adults who had received computer and internet training were interviewed about their attitudes and experiences towards the use of internet as a part of the Care OnLine project. Most of these older adults (82%) reported that the use of the internet had a positive impact on their QoL. Using the internet decreased isolation and improved interactions socially in this study (Osman *et al.*, 2005). Thirty-three older adults interviewed in a study in China reported that using the internet was helpful in making their lives after retirement more meaningful and improving their self-evaluation. It was concluded by the author that using the internet resulted in a sense of well-being in these older adults (Xie, 2007). Koopman-Boyden and Reid (2009) also reported a positive correlation between the use of internet by older adults and their well-being. An analysis of a subset of the European Social Survey (ESS1) showed that older adults (*n* = 11,000) using the internet regularly had decreased social isolation and better life satisfaction. The older adults in the study reported that communicating using the internet helped and was complementary to face-to-face interactions (Lelkes, 2012). Chaumon *et al.* (2014) found that in 17 older adults living in long-term care with a history of functional loss, the use of computers improved the QoL in general and additionally had a positive impact on their self-sufficiency, self-efficiency and psychological empowerment. In quantitative surveys (*n* = 1780) in older adults (60 years and older), it was seen that information and communication technology use contributes to their well-being (Ihm and Hsieh, 2015).

Some studies have found conflicting results, for example, in their study, Firth and Mellor (2009) did not find a significant difference in the older adults who were using internet for the last 12 months in terms of quantitative scores compared with older adults who were not using the internet. Conversely, when interviewed the older adults using the internet reported a better social connectedness and had a better understanding of using the internet for new learning opportunities. Similarly, in another study, at the end of 12-month training on internet use, 20 older adults when interviewed reported positive experiences and attitudes associated with the use of the internet even though the reported well-being scores in the study did not show improvements (Mellor *et al.*, 2008). That said, there are studies which did not show either a positive impact or any correlation between the use of internet by older adults and life satisfaction, QoL or well-being. On analysis of the data from National Health and Aging Trends Study (*n* = 6443), the authors did not find any correlation of information and communication technology use with well-being of older adults using the internet (Elliot *et al.*, 2013). Similarly, in another study, life satisfaction was similar between older adults who were a part of an online training and those who were not (Dorin, 2007).

## Critical analysis summary of the studies

The studies looking at use of the internet in the older adults are seeing an increase in the last decade. More research is focused on the acceptance of internet technology, the barriers to use of internet technology and the uses of internet technology. Little research focuses on impact on QoL. As it is summarised in Table 1, only one of the studies used a validated QoL scale, the WHOQoL, and one used the SF-36 while none of the studies used the CASP-19. In the studies reviewed, there was a mix of studies looking at older adults who had never used the internet before and were given training during the course of the study, while other looked at older adults using the internet for some time. This could be one of the reasons for discordance between the results on impact of internet use on factors associated with well-being, life satisfaction and other parameters. Many of the studies did not use validated software like the SPSS for quantitative analysis or NVivo for qualitative analysis. Very few studies use a mixed methods approach, and the sample size for most studies was small. Research from developing countries is still lacking. All the studies reviewed here related to impact of internet use on older adults were done in developed countries except for one in China. The findings of this review point towards important policy and practice approaches including exploring investing in Information and communications technologies (ICT) training in pre-retirement courses in public and private sector organisations and subsidies for ICT purchase or one-to-one guidance on ICT use.

## Conclusion

On review of the published studies, there is an indication that the use of internet by older adults helps to improve life satisfaction and well-being. The majority of the studies substantiate the advantages of internet use by older adults including the ability to communicate with family and friends, maintain a wide social network, have access to information and participate in online leisure activities. There are some studies, though less in number, which did not find a relationship between well-being and use of internet by older adults. This could possibly be attributed to a ‘ceiling effect’ as the scores of the participants in some of these studies were already high at baseline, so the scope of improvement was attenuated (Chen and Persson, 2002; White *et al.*, 2002) or possibly due to the bias in these studies of not separating the use of the internet from internet training (Slegers *et al.*, 2008). It was also seen that studies with a qualitative methodology were more likely to elicit a positive impact of the use of internet than quantitative studies.

The gap areas identified from the literature review include the paucity of research in this area from developing countries, few studies with a mixed methods research methodology, the sample size in the studies which is usually small, studies limited to older adults in 60 years and above and only a limited number of studies used a validated QoL score to study the impact of internet use by older adults on QoL.
